# Effects of different ages on frozen semen quality and *in vitro* fertilization efficiency in Wannan black pigs

**DOI:** 10.3389/fvets.2024.1395718

**Published:** 2024-05-31

**Authors:** Changzhi Xu, Xianshu Yang, Heming Sui, Xu Tong, Dandan Zhang, Xianrui Zheng, Jun Jiao, Chonglong Wang, Zubing Cao, Yunhai Zhang

**Affiliations:** ^1^Anhui Province Key Laboratory of Local Livestock and Poultry, Genetical Resource Conservation and Breeding, College of Animal Science and Technology, Anhui Agricultural University, Hefei, China; ^2^Suzhou Key Laboratory of Reproductive Medicine, Department of Reproductive Medicine, General Hospital of WanBei Coal Group Hospital of WanBei Coal Group, Suzhou, China; ^3^Anhui Haoyu Animal Husbandry Co. Medicine, Anhui Academy of Agricultural Sciences, Hefei, China; ^4^Institute of Animal Husbandry and Veterinary Medicine, Anhui Academy of Agricultural Sciences, Hefei, China

**Keywords:** pig, aging, frozen semen, sperm quality, fertility

## Abstract

According to previous studies, the quality and fertilization rate of fresh sperm from boars of different ages were significantly different. However, the difference of freeze–thaw sperm quality and fertility in boars of different ages is unclear. In this study, boars of a Chinese native breed were assigned into two groups. Each group consisted of five boars aged aged either 2–3 years (young boars = YB) or 5–6 years (aging boars = AB) A total of 60 ejaculates for each group were collected and cryopreserved. Semen quality and *in vitro* fertility of post-thaw sperm was evaluated. The results showed that the concentration and motility of fresh sperm collected from AB were similar to YB, but their semen volume was higher than that in YB (*p* < 0.05). Frozen–thawed sperm of AB had lower viability than YB, and higher abnormal rate and reactive oxygen species (ROS) levels of YB (*p* < 0.05). There was no effect of the age on post-thaw sperm motility and time survival. Functional assessments indicated that increasing age markedly compromises the integrity of the sperm plasma membrane and acrosome, as well as mitochondrial functionality post-thaw, albeit without affecting DNA integrity. Furthermore, increasing age of boars reduces the ability of sperm to bind to the oocyte zona pellucida after thawing, delaying the time of the first embryo cleavage after fertilization. Finally, the early developmental efficiency of *in vitro* fertilized embryos progressing from 4-cell to blastocyst derived from post-thaw sperm in AB significantly decreased compared to those from YB (*p* < 0.05). Taken together, these results suggest that increasing age in boars impairs the quality and *in vitro* fertility of frozen thawed sperm.

## Introduction

Cryopreservation of sperm has an important significance in both the protection of genetic resources and the highly efficient utilization of semen in animal and human artificial insemination practices (1). However, the quality of frozen–thawed boar sperm and the conception and litter size of sows post-insemination are generally inferior to those of liquid-stored semen (2, 3). It is reported that the quality and fertility of post-thaw sperm are usually affected by the inherent physiological conditions of boars *per se* and physical and chemical stresses during freezing–thawing processes (4). Previous studies indicate that the quality and fertility of semen are tightly correlated with boar ages. Hensel found that ejaculate volume was lower in boars under 18 months of age than in boars 18 to 36 months of age, and > 36 months of age, but semen density was significantly higher in boars under 18 months of age than in boars 18 to 36 months of age, and > 36 months of age (5). Damian Knecht’s teamfound that boar ejaculate volume increases with age, but semen density decreases significantly, and suggested that the optimal age for boar production performance is 19–24 months of age (6). Also, studies comparing the reproductive performance of artificially inseminated boars with that of sows have shown that the age of the boar also affects conception rates and the number of live births (7). Thus, the accurate selection of boar suitable ages before semen collection is critical for the production of high-quality sperm.

Within the physiological framework, the reproductive viability of boars is inherently finite. Typically, boars commence breeding activities at approximately 2 years of age, with their lifespan generally extending to about 5 to 6 years. Boars enter adolescence around 7–8 months, achieving sexual maturity as they progress through development stages (8). The term “reproductive performance” of boars is defined as their capacity to sustain normal reproductive functions and produce viable offspring over a specific timeframe. Conventionally, the peak of boars’ reproductive performance ceases around 36 months of age, although in subtropical climates, this period may prolong to as much as 51 months (9). Once boars age beyond reproductive longevity, sperm function and fertility gradually decline. Indeed, numerous studies have suggested that aging has negative effects on the quality and fertility of fresh sperm in several species. For instance, aging severely reduced semen quality and caused infertility in humans (10) and mice (11). Advanced age altered the biochemical composition of boar semen (12) and impaired the genomic stability and quality of boar sperm (13). In rams and bulls, aging caused lower semen quality and abnormal semen composition (14, 15). Paternal aging also delayed the development of progeny in bustards (16) and reduced the semen production of offspring in birds (17). Evidence also suggests a discernible decline in the quality of frozen–thawed sperm from aged dogs (18, 19). Despite these findings, the specific effects of boar aging on the quality and fertility of frozen–thawed spermatozoa require further elucidation.

The present study was conducted to investigate the effects of different ages on the quality of fresh and frozen–thawed semen, along with the *in vitro* fertility. In addition, increasing age delays the rate of the first cleavage of the fertilized egg and reduces the early development potential of the embryo after thawing. The results of this study could be helpful to select suitable age boars for production of frozen semen and expand the application of frozen semen in the pig industry.

## Materials and methods

All experiments using boars were conducted in accordance with the Institutional Animal Care and Use Committee (IACUC) guidelines under current approved protocols at Anhui Agricultural University.

### Semen collection and concentration analysis

Ten healthy Wannan black boars (a Chinese native pig breed) were distributed into two age groups. All test boars were sampled in the same rearing environment and test conditions, with the only variable being age, which ranged from 2 to 3 years in the younger group and 5–6 years in the older group. Five boars in each group were raised under the same management conditions with controlled environment temperature, fed with the same diets, exposed to a total of 16 h nature plus artificial light, and given *ad libitum* access to water. Sperm-rich fractions were collected using the gloved hand method. Semen was collected for each boar twice a week and six ejaculates per boar (60 ejaculates in total) were used for sperm freezing and quality evaluation. The sample volume was measured by means of a measuring cylinder. The concentration of fresh sperm was evaluated using a commercial computer assisted sperm analysis (CASA) system (SQA-ZXZ-8001, ZhuXianZi, Guangzhou, China).

### Freezing and thawing of semen

Semen free of foreign matter, without noticeable odor, milky white in color, and with sperm viability greater than 90% was selected for freezing. Fresh semen was stored at 17°C to cool for 2 h, then centrifuged at 800 × *g* for15 min. Freezing is done using a two-step process. Following the removal of seminal plasma, sperm pellets were diluted to 2 × 10^9^ sperm/mL in a commercial cooling medium (Green Auris, Beijing, China) containing 20% egg yolk. Then, the sperm cells were cooled slowly to 4°C for 3 h and diluted to 1 × 10^9^ sperm/mL with a commercial freezing medium (Green Auris, Beijing, China) at 4°C. Subsequently, sperm samples were packed in 0.5 mL labeled plastic straws (Minitube, Germany) and the straws were sealed. The straws were then frozen by exposure to liquid nitrogen vapor at 3 cm above the liquid nitrogen level for 10 min. The straws were finally plunged into liquid nitrogen and stored. The frozen straws were thawed by immersion in a water bath at 50°C for 15 s.

### Determination of sperm quality

The quality of fresh and thawed sperm was evaluated using a CASA system with a disposable sperm counting chamber at 37°C (ZhuXianzi, Guangzhou, China). Sperm motility and viability were obtained by CASA system. The fresh semen was diluted 1/1000 (v:v) in a commercial extender (Androhep® Plus, Minitube, Germany). The thawed semen was diluted 1:10 (v:v) in a commercial extender (Green Auris, Beijing, China). For frozen–thawed semen, the analyses were carried out in duplicate (two straws for each treatment).

### Measurement of sperm survival time

Sperm survival time was assessed by thawing frozen boar semen. After thawing, the frozen fine tubes were cut open and placed in 1.5 mL centrifuge tubes, and 10 μL of the sample was placed under the CASA system microscope for observation, and sperm activity was observed with a clock timer, which was stopped when there was no sperm activity.

### Assessment of sperm viability

After thawing, 50 μL semen was taken and mixed with 1 mL of 0.4% trypan blue in 0.9% NaCl and then incubated for 15 min at 37°C. Viable spermatozoa remained unstained while dead cells were stained blue. The percentage of unstained spermatozoa was calculated in a sample of 200 spermatozoa under 400X magnification.

### Evaluation of sperm morphology

The morphological abnormalities of spermatozoa were assessed using eosin staining (Solarbio, E8090). After thawing, each sample was smeared on a clean glass slide, air-dried and stained for 15 min. For each slide, 200 spermatozoa were evaluated by a 400X light microscope.

### Evaluation of sperm plasma membrane integrity

The membrane integrity was analyzed using the hypo-osmotic swelling test (HOST). One mL of semen and 10 mL hypo-osmotic solution [7.35 g of sodium citrate (Genuine Leaf Bio, S11111) and 13.51 g of fructose (Sigma, F3501) in 1000 mL of distilled water] was mixed. After incubation for 30 min at 37°C, sperm swelling was assessed by placing 10 μL well-mixed samples on a warm slide (37°C) under a phase-contrast microscope at 400 × magnification. A total of 300 spermatozoa per slide were counted in at least three different microscopic fields. The spermatozoa were classified as positive or negative based on the presence or the absence of coiled tail. The percentages of sperm with swollen and curled tails were then recorded.

### Evaluation of sperm acrosome integrity

The 20 μL semen was used to prepare smears on microscope slides. After air-drying, sperm smears were fixed with absolute methanol for 10 min at room temperature and allowed to dry. Then, approximately 20 μL FITC-labeled peanut agglutinin (FITC-PNA) solution (Sigma, L7381) in PBS was spread over each slide. Subsequently, the slides were incubated in a dark and moist chamber for 30 min at 37°C. After incubation, the slides were rinsed with PBS and air-dried. The slide smear was covered by slip and sealed with colorless nail polish. The acrosome status of the sperm was examined using an epifluorescence microscope (Olympus, Tokyo, Japan). The whole acrosome was visualized with strong green fluorescence under a fluorescence microscope and was scored as acrosome-intact spermatozoa. The percentage of acrosome-intact spermatozoa was calculated in at least 200 sperm cells per slide.

### Evaluation of sperm mitochondrial function

Mitochondrial activity of spermatozoa was assessed using Rhodamine 123 (Nanjing Pars Biotechnology)/propidium (Aladdin) (Rh123/PI) dual fluorescent staining. The 100 μL PBS was preheated at 37°C for 10 min, then mixed with 1 μL PI and 1 μL Rh123, and incubated for 10 min in the dark at 37°C. Next, 50 μL semen sample was added in staining solution and incubated at 37°C for 30 min in the dark and humid environment. Following incubation, 10 μL semen sample was taken onto a slide and covered with a coverslip and sealed with colorless nail polish. The fluorescence intensities of sperms were examined using an epifluorescence microscope (Olympus, Tokyo, Japan). Spermatozoa displaying a green tail and a red head were scored as normal mitochondrial function. Spermatozoa exhibiting only a red head indicated dead sperms. The percentage of spermatozoa with normal mitochondrial function was counted in at least 200 sperms per slide.

### Evaluation of sperm DNA integrity

The 10 μL semen sample was taken onto a slide and dried at room temperature. Spermatozoa were fixed in anhydrous ethanol-glacial acetic acid (3:1) for 5 min and stained with freshly prepared acridine orange solution for 10 min. After washing and drying, the DNA status of sperms was examined using an epifluorescence microscope at 400 × magnification (Olympus, Tokyo, Japan). Spermatozoa exhibiting a green head were indicated as intact DNA. The percentage of spermatozoa with intact DNA was counted in at least 200 sperms per slide.

### Assessment of sperm ROS levels

ROS levels of spermatozoa were measured using DCF assay. DCFH-DA solution (Beyotime, S0033S, China) was added to spermatozoa at 1 × 10^6^ cells/mL and incubated for 15 min at 37°C. The fluorescence intensities of sperms were examined using an epifluorescence microscope (Olympus, Tokyo, Japan) with excitation and emission wavelengths of 488 and 525 nm, respectively.

### *In vitro* maturation of oocytes

Ovaries were collected from a local slaughterhouse. Follicular fluid was aspirated from antral follicles at 3–6 mm in diameter. Cumulus-oocyte complexes (COCs) were selected under a stereomicroscope. Subsequently, COCs were cultured in one well of 4-well plate containing 400 μL *in vitro* maturation medium [TCM-199 (Sigma, M4530) supplemented with 5% FBS, 10% porcine follicular fluid, 10 IU/mL eCG (NSHF), 5 IU/mL hCG (NSHF), 100 ng/mL L-Cysteine (Sigma), 10 ng/mL EGF (Sigma), 0.23 ng/mL melatonin, 2.03 × 10–5 ng/mL LIF, 2 × 10–5 ng/mL IGF-1, 4 × 10–5 ng/mL FGF2, 100 U/mL penicillin and 100 mg/mL streptomycin] for 44 h at 38.5°C, 5% CO_2_ and saturated humidity. Cumulus cells surrounding oocytes was removed using 1 mg/mL hyaluronidase following maturation.

### *In vitro* fertilization

Matured oocytes were washed in the modified Tris-buffered medium (mTBM) containing 2 mg/mL BSA and 2 mM caffeine. Approximately 15 oocytes were incubated in 50 μL droplets of mTBM for 4 h at 38.5°C in 5% CO2 in air. To prevent individual differences, semen from two boars was mixed and centrifuged at 1900 *g* for 4 min in DPBS supplemented with 1 mg/mL BSA (pH 7.3). Then, sperms were resuspended with mTBM to a concentration of 1 × 10^6^ cells/mL. Fifty microliters of the sperm solution was added to the mTBM droplets containing oocytes. After co-incubation of oocyte and sperm for 6 h, sperms surrounding oocytes were washed out and presumptive zygotes were cultured in PZM-3 at 38.5°C in 5% CO_2_ in air. Following *in vitro* fertilization, the number of embryos cleaved at 24 h, 30 h, 48 h and blastocysts was recorded using a microscope (Olympus, Tokyo, Japan).

### Sperm binding analysis

Spermatozoa were co-incubated with oocytes for 6 h. The oocytes were fixed in 4% paraformaldehyde for 30 min, followed by staining with Hoechst (10 mg/ml). The number of bound sperms surrounding each oocyte was counted from z projections acquired by the confocal microscope (Olympus, Tokyo, Japan).

### Statistical analysis

Statistical analysis was performed using independent samples t-test analysis (SPSS 17.0). Percentage data were transformed with an arcsine before statistical analysis. All experiments were performed at least three times and expressed as mean ± standard error (mean ± S.E.M.). *P* < 0.05 was considered to be statistically significant.

## Results

### Differences in fresh semen quality of local boars of different ages

**Figure 1:**
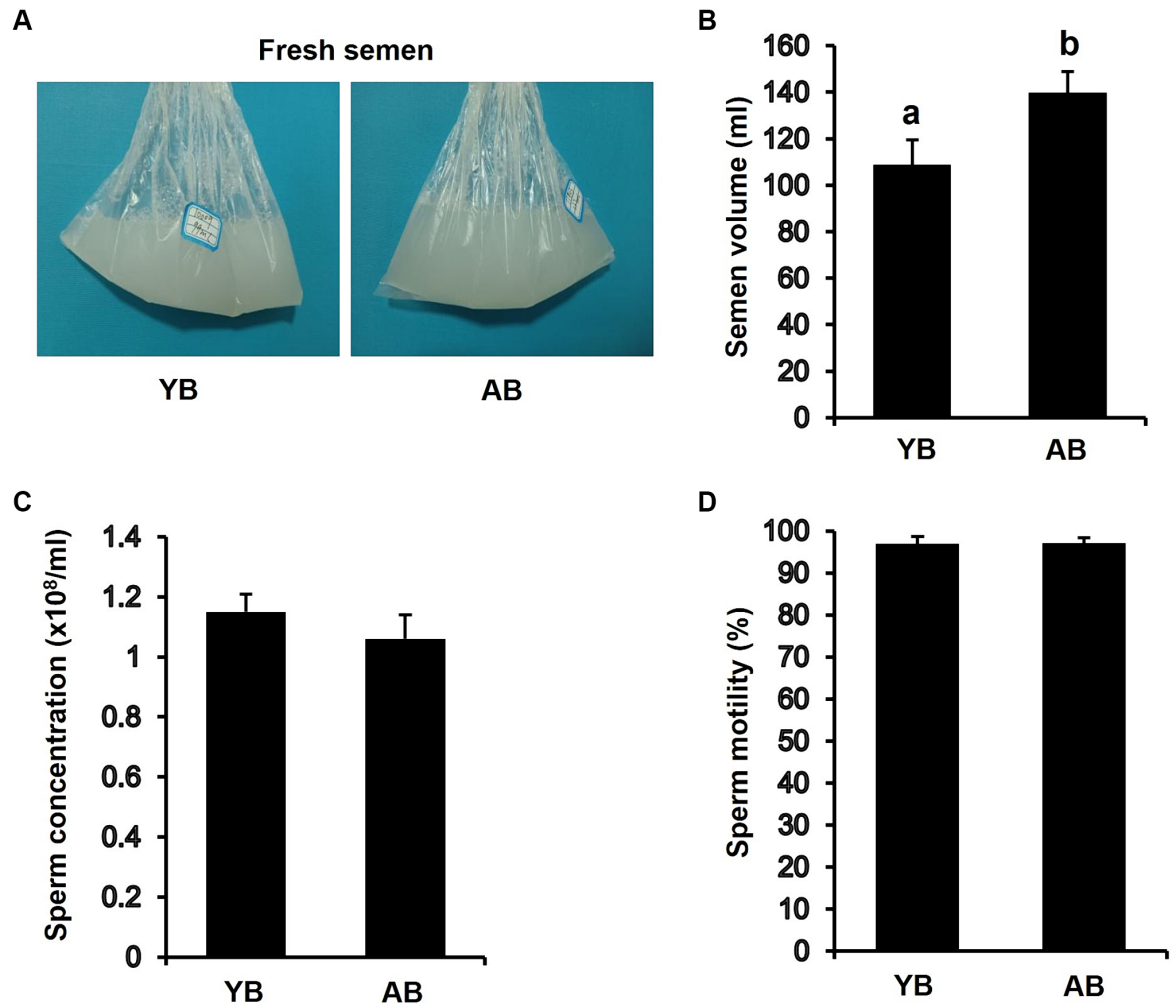
Analysis of fresh semen quality between young and aged boars. **(A)** Representative images of semen collected from YB and AB. YB, young boar; AB, aged boar. **(B)** Comparison of semen volume per ejaculation between YB and AB. **(C)** Sperm concentration of fresh semen between YB and AB. **(D)** Sperm motility of fresh semen between YB and AB. All data are shown as mean ± S.E.M and different letters on the bars indicate significant differences (*p* < 0.05).

To determine whether semen quality in aged boars (AB) is similar to that in young boars (YB), several parameters of fresh semen were analyzed. As shown in Figure 1A, all color of semen from AB and YB was milk white. We observed that the semen volume of AB was significantly higher than that in the YB (Figure 1B; *p* < 0.05). However, sperm concentration (Figure 1C) and motility (Figure 1D) of AB had no significant difference compared to those in the YB. Thus, these results suggest that the effect of increasing age on fresh semen of local boars was not significant.

### Differences in the quality of frozen and thawed spermatozoa of local boars of different ages

**Figure 2:**
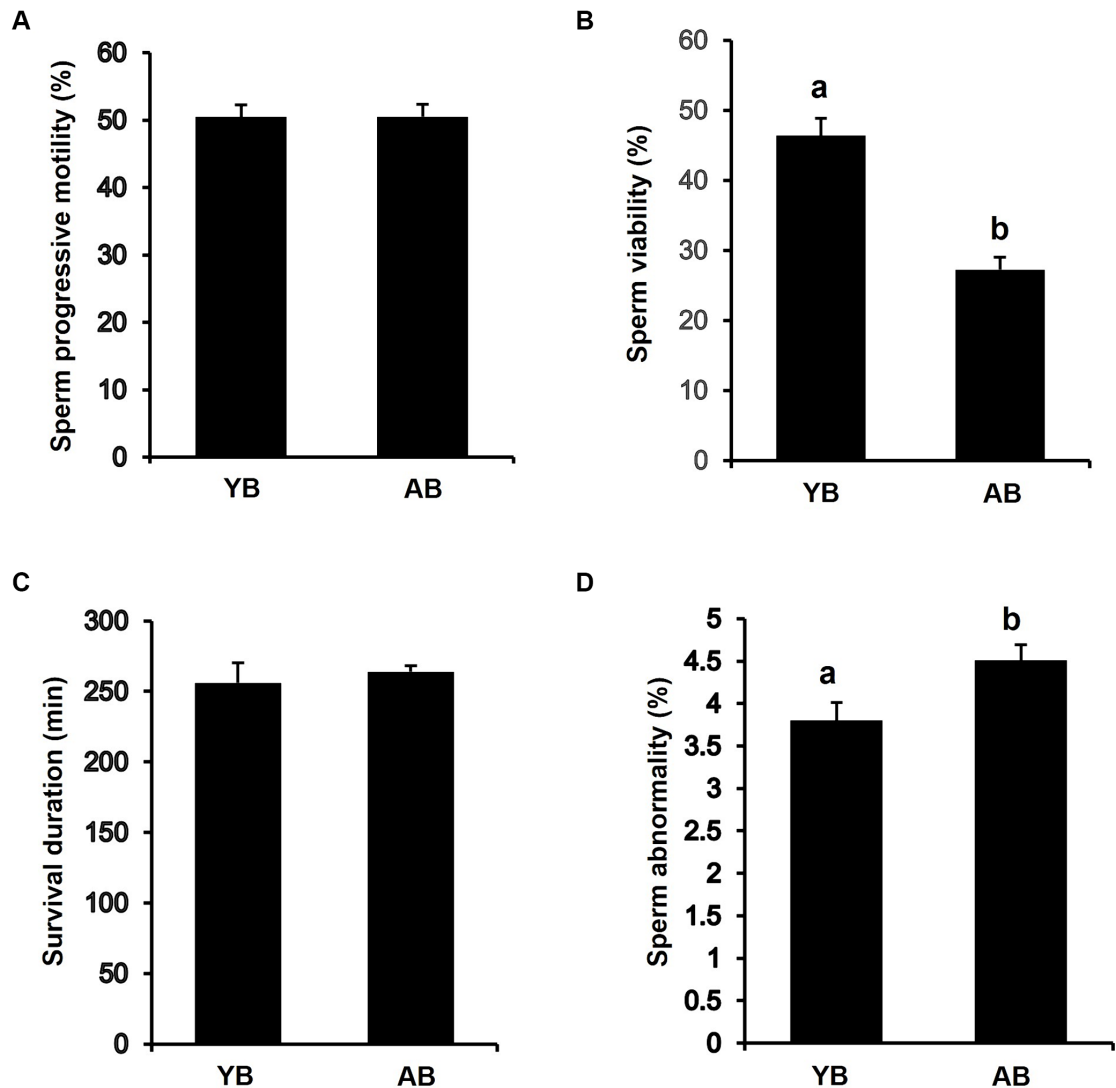
Analysis of frozen–thawed sperm quality between young and aged boars. **(A)** Progressive Motility of frozen–thawed sperm between YB and AB. **(B)** Viability of frozen–thawed sperm between YB and AB. **(C)** Survival duration of frozen–thawed sperm between YB and AB. **(D)** Abnormality rate of frozen–thawed sperm between YB and AB. All data are shown as mean ± S.E.M and different letters on the bars indicate significant differences (*p* < 0.05).

To determine whether increasing age affects the quality of frozen thawed sperm, we analyzed the progressive motility, viability, survival time, and abnormal sperm rate of thawed sperm from boars of different ages. The results showed that progressive motility and survival duration of post-thaw sperm from AB were similar to those in YB (Figures 2A,C). However, the viability of post-thaw sperm from AB was significantly lower than that in YB (Figure 2B; *p* < 0.05). Besides, the rate of morphologically abnormal sperm from AB was significantly higher than that in YB (Figure 2D; *p* < 0.05). Thus, these results reveal that the frozen–thawed spermatozoa in AB are inferior in quality to those in YB.

### Differences in functional characteristics of frozen–thawed spermatozoa of local boars of different ages

**Figure 3:**
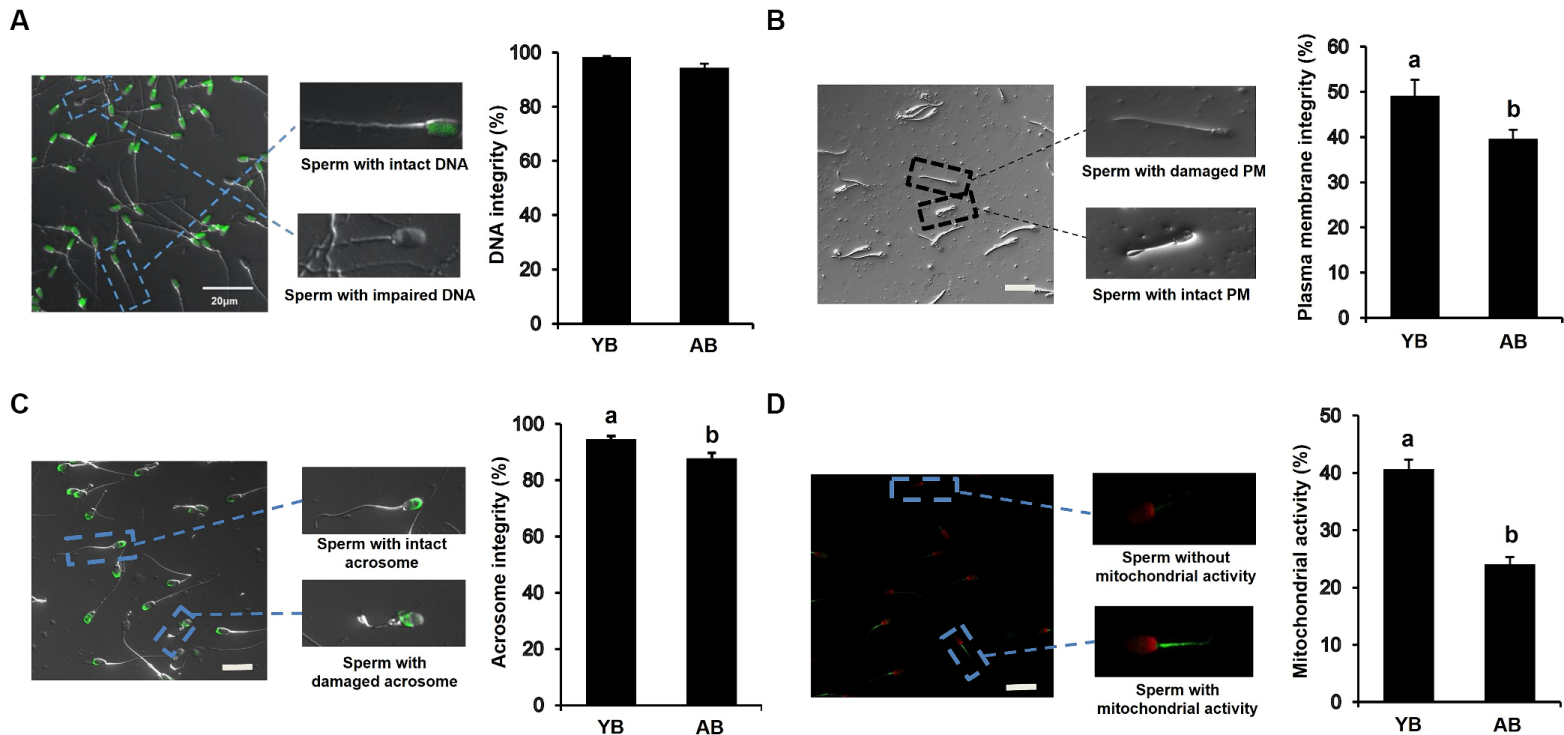
Functional characteristics of frozen–thawed sperm between young and aged boars. **(A)** DNA integrity of frozen–thawed sperm between YB and AB. **(B)** Plasma membrane integrity of frozen–thawed sperm between YB and AB. **(C)** Acrosome integrity of frozen–thawed sperm between YB and AB. **(D)** Mitochondrial activity of frozen–thawed sperm between YB and AB. Dash squares indicate representative spermatozoa. All data are shown as mean ± S.E.M and different letters on the bars indicate significant differences (*p* < 0.05).

To examine whether the increase of age affects the functional activity of frozen–thawed sperm, the functional characteristics of thawed spermatozoa were analyzed. As shown in Figure 3A, there was no significant difference in DNA integrity of post-thaw sperm between AB and YB. However, we found that the percentages of post-thaw sperm with intact plasma membrane and acrosome, and normal mitochondrial activity in AB significantly reduced compared to those in YB (Figures 3B–D; *p* < 0.05). Furthermore, the ROS levels of post-thaw sperm in AB were apparently higher than those in YB (Supplementary Figures S1A,B; *p* < 0.05). Together, these data demonstrate that the increase in age seriously impairs the functional characteristics of frozen and thawed pig sperm.

### Differences of oocyte binding ability of frozen–thawed sperm and first cleavage time of embryos after fertilization in boars of different ages

**Figure 4:**
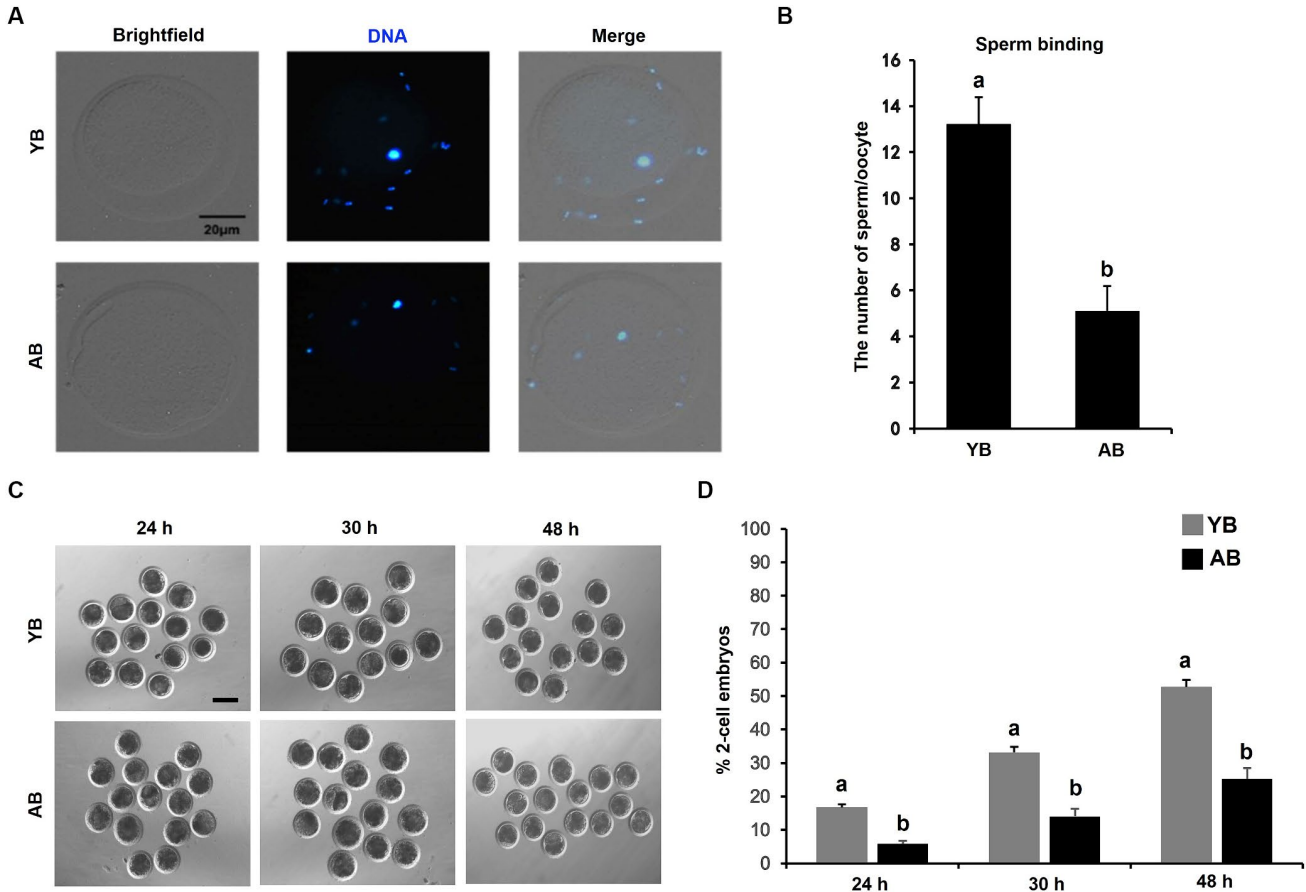
Comparison of oocyte binding ability and fertilization potential of frozen–thawed sperm between young and aged boars. **(A)** Representative images of oocytes bound by frozen–thawed sperm. Oocytes with pb1 extrusion were incubated with capacitated sperm for 6 h to carry out the oocyte binding assay. The experiment was replicated 5 times and 150 oocytes in each boar was analyzed. Scale bar: 20 μm. **(B)** The number of frozen–thawed sperm binding to the surface of zona pellucida surrounding oocytes. **(C)** Representative images of embryos cleaved at different timing points. Scale bar: 100 μm. **(D)** The cleavage rates of oocytes fertilized by frozen–thawed sperm between YB and AB. All data are expressed as mean ± S.E.M and different letters on the bars indicate significant differences (*p* < 0.05).

To investigate whether the increase of age will impair the binding ability of frozen–thawed sperm to oocytes, the Hoechst staining of oocytes fertilized by post-thaw sperm was carried out. As presented in Figures 4A,B, the number of YB post-thaw sperm bound to zona pellucida of oocytes was much more than that in AB (*p* < 0.05). The timing of the first embryonic cleavage post-fertilization was further analyzed. The results revealed that the cleavage rates of embryos at post-fertilization 24 h, 30 h and 48 h in YB were higher than those in AB (Figures 4C,D; *p* < 0.05). Therefore, these data show that the increase in age reduces the oocyte binding ability of frozen–thawed boar spermatozoa and the first division after fertilization.

### Differences in early development efficiency of frozen–thawed sperm *in vitro* fertilized embryos of boars of different ages

**Figure 5:**
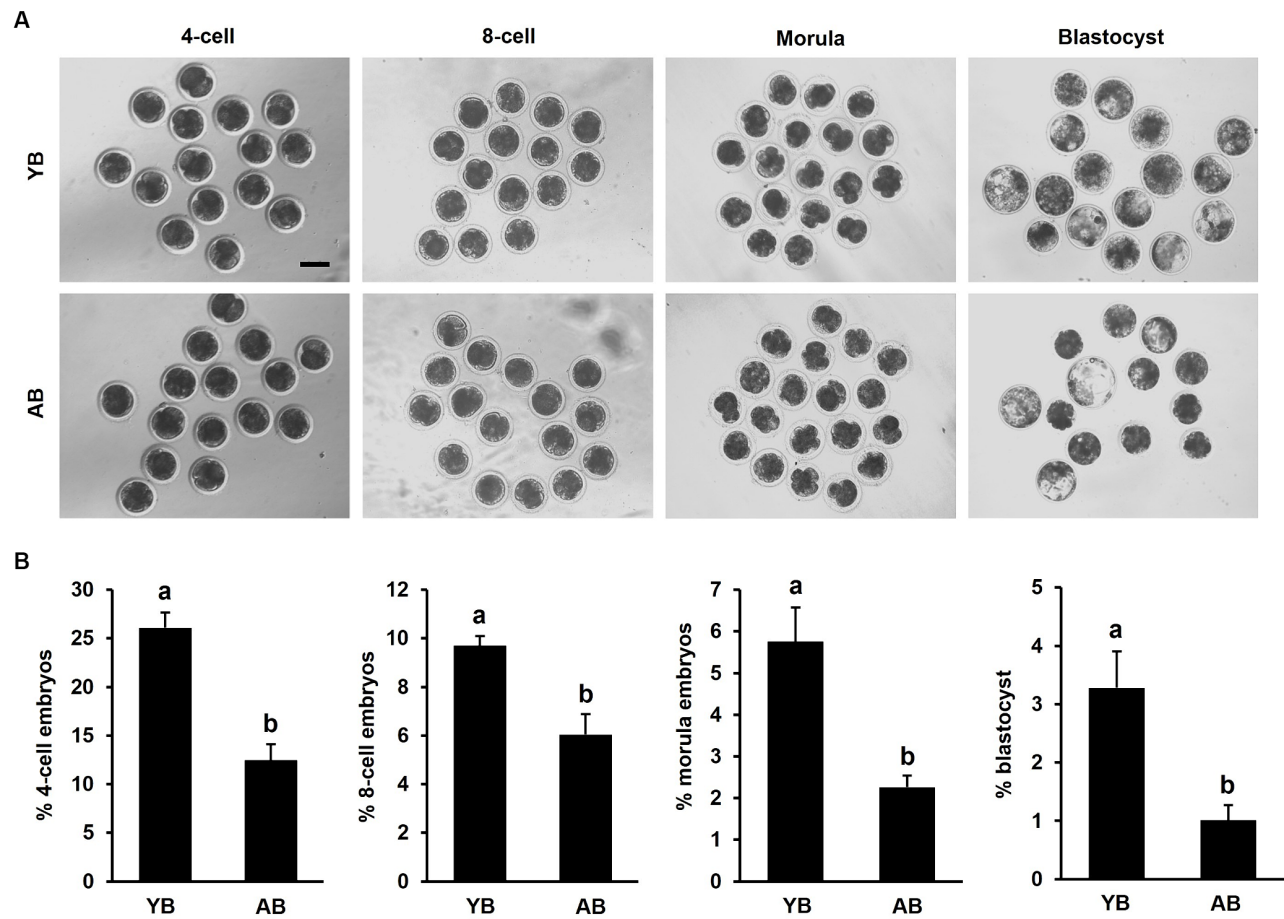
Developmental efficiency of *in vitro* fertilized embryos produced by frozen–thawed sperm between young and aged boars. **(A)** Representative images of early embryos derived from frozen–thawed sperm between YB and AB. The experiment was replicated 5 times. A total of 415 or 417 oocytes in young or aged boars was analyzed, respectively. **(B)** Developmental rates of 4-cell, 8-cell, morula, and blastocyst between YB and AB. All data are expressed as mean ± S.E.M and different letters on the bars indicate significant differences (*p* < 0.05).

To further study whether the increase of age reduced the developmental competence of early embryos derived from frozen–thawed sperm, the number of embryos from 4-cell to blastocyst stage was recorded. The results indicated the developmental rates of embryos from 4-cell to blastocyst derived from post-thaw sperm in AB were significantly lower than those in YB (Figures 5A,B; *p* < 0.05). Therefore, these results document that the increase of age decreases early developmental potential of *in vitro* fertilized embryos produced by frozen–thawed sperm.

## Discussion

Extant literature has established that the advancement in boar age influences the quality of fresh semen (5), nevertheless, the implications of age increment on the quality and fertilization efficacy of frozen–thawed boar spermatozoa remain uncertain. To elucidate this issue, the current study utilized a local Chinese boar breed as a model. Our findings reveal that while the aging of boars does not alter the concentration and motility of fresh semen, it does modify the functional parameters of frozen–thawed spermatozoa. Consequently, these changes lead to a diminished capacity for early developmental success in embryos derived from thawed sperm.

The role of advancing age as a determinant of reproductive longevity in male animals has been substantiated in previous research (10, 20). Studies have shown that natural male aging could dramatically reduce the quality and composition of fresh semen in humans (21), rams (14), bulls (15), and dogs (19). However, it is still controversial whether the increase of age will have a negative impact on the quality of fresh pig semen. Some studies have reported that the increase in boar age can damage the physiological function and biochemical composition of fresh sperm (12, 13). Consistent with another study (22), our results show that age does not change the concentration and motility of fresh sperm in local boars. Further complicating the discourse, evidence from research on bulls suggests that older males may exhibit increased semen volumes compared to their younger counterparts (23–25). This increment in semen volume has been linked to the maturation of parasympathetic glands, enhanced scrotal circumference, and overall weight gain (26, 27). Mirroring these findings, the present study discerns that older boars exhibit a significant increase in semen release, both in volume and concentration, an observation that might be attributed to the altered accessory gonadal secretion functions in aged local boars. This research not only contributes to the ongoing discourse regarding the effects of aging on semen quality but also underscores the complexity of reproductive aging dynamics in male animals, particularly in porcine species.

The viability and functional integrity of frozen–thawed spermatozoa are frequently compromised by intrinsic adverse factors associated with the male animals from which they originate, particularly where the phenomenon of age-related physiological stress is implicated. The functional parameters of post-thaw sperm can reflect its quality. Emerging evidence, for instance, from studies on canine species, has pointed out a significant diminution in both total and progressive motility (19) as well as acrosome integrity (18) as a consequence of male aging. Analogously, in porcine research, it has been elucidated that aging during extended periods of cryopreservation leads to a conspicuous decline in post-thaw sperm plasma membrane integrity and motility (28, 29). Corroborating these studies, our investigation reveals that aging in local boars markedly compromises the plasma membrane and acrosome integrity of sperm. However, in this study the increase in age did not alter the post-thaw sperm motility. The discrepancy could be attributed to the species or breed differences.

Mitochondria are implicated in maintaining ROS levels at the physiological levels (30). A balance between ROS levels and antioxidant defense is needed to ensure normal cellular functions. Research has demonstrated that advancing age can perturb the redox balance within sperm mitochondria (31), leading to mitochondrial dysfunctions and consequent overproduction of reactive oxygen species (ROS) in sperm (32). The present study demonstrated a significant reduction in mitochondria activity and increased ROS levels of post-thaw sperm in aged boars. Similarly, mitochondrial activity is associated with sperm motility performance. Research has demonstrated the pivotal role of mitochondrial integrity and function in spermatozoa (33). Boar spermatozoa particularly rely on mitochondrial ATP for motility (34); thus, a diminished mitochondrial membrane potential (MMP) can impede spermatozoa viability. Consequently, alterations in MMP serve as a promising indicator of impaired functionality (35). Thus, aging may impair mitochondrial function, which in turn induces high ROS levels in thawed spermatozoa as well as motility dysfunction. Furthermore, numerous studies have shown that oxidative stress created by excessive ROS caused poor sperm quality in different species (32), which could account for aging-induced functional defects of post-thaw sperm in local boars.

Male fertility is determined to a large extent by the quality of sperm. The fertilizing potential and fertility are golden criteria for evaluating the post-thaw sperm quality. Previous studies have underscored the importance of the paternal contributions of sperm to early embryo development (36, 37). It has been found that aging impaired the fertilizing competence of fresh sperm and the developmental capacity of early embryos in mice (11) and humans (38). The results of the present investigation corroborate these findings, revealing that the aging process in boars significantly compromises the fertilization capacity of post-thaw sperm, as well as the early developmental efficacy of embryos, and induces a deceleration in the cleavage kinetics of zygotes. Therefore, it can be posited that the diminished quality of post-thaw sperm is a contributing factor to the observed decline in fertilization potential and *in vitro* fertility in local boar populations. This underscores the necessity for future research to conduct comparative analyses of cryopreserved semen quality and *in vitro* fertilization efficiency across boars of varying age groups, thereby providing deeper insights into the age-related dynamics influencing reproductive success in these animals.

In conclusion, the findings of this study demonstrate that advancing age in native boars detrimentally impacts the quality and *in vitro* fertility of cryopreserved spermatozoa. Such insights are instrumental in enhancing the production efficiency of boar cryosemen and facilitating its expanded use in the artificial insemination of swine. This investigation underscores the significance of considering age-related variables in the optimization of cryopreservation protocols and artificial insemination strategies, thereby contributing to the improvement of reproductive outcomes in pig breeding programs. The present study was an exploration of semen from a small number of Chinese endemic pigs, which may limit the generalizability of the findings.

## Data availability statement

The original contributions presented in the study are included in the article/Supplementary material, further inquiries can be directed to the corresponding authors.

## Ethics statement

The animal study was approved by the Ethics Committee of Anhui Agricultural University under permit No. AHAU 20101025. The study was conducted in accordance with the local legislation and institutional requirements.

## Author contributions

CX: Methodology, Writing – review & editing, Formal analysis, Investigation, Validation. XY: Formal analysis, Validation, Writing – review & editing, Software. HS: Formal analysis, Validation, Writing – review & editing, Investigation, Methodology. XT: Investigation, Writing – original draft. DZ: Investigation, Writing – original draft. XZ: Writing – original draft, Writing – review & editing. JJ: Writing – original draft, Resources. CW: Writing – original draft, Writing – review & editing. ZC: Writing – original draft, Writing – review & editing, Conceptualization, Funding acquisition, Methodology, Project administration, Supervision. YZ: Conceptualization, Data curation, Formal analysis, Funding acquisition, Investigation, Methodology, Project administration, Resources, Software, Supervision, Validation, Visualization, Writing – original draft, Writing – review & editing.

## Supplementary material

FIGURE S1 Analysis of ROS levels of frozen-thawed sperm between young and aged boars. **(A)** Representative images of ROS staining in frozen-thawed sperm between YB and AB. **(B)** ROS levels in frozen-thawed sperm between YB and AB. The fluorescence intensity of ROS was measured The fluorescence intensity of ROS was measured by the confocal microscopy using identical settings and parameters. The data are presented as mean ± S.E.M and different letters on the bars indicate significant differences (*p* < 0.05). The data are presented as mean ± S.E.M and different letters on the bars indicate significant differences (*p* < 0.05).

## References

[1] Yanez-Ortiz I Catalan J Rodriguez-Gil JE Miro J Yeste M. Advances in sperm cryopreservation in farm animals: cattle, horse, pig and sheep. Anim Reprod Sci. (2022) 246:106904. doi: 10.1016/j.anireprosci.2021.106904, PMID: 34887155

[2] Funahashi H. Methods for improving in vitro and in vivo boar sperm fertility. Reprod Domest Anim. (2015) 50:40–7. doi: 10.1111/rda.12568 26174918

[3] Yeste M Rodriguez-Gil JE Bonet S. Artificial insemination with frozen-thawed boar sperm. Mol Reprod Dev. (2017) 84:802–13. doi: 10.1002/mrd.22840 28608609

[4] Bingyu Bai CX Wen Y Lim J Le Z Shou Y Shin S. Cryopreservation in the era of cell therapy: revisiting fundamental concepts to enable future technologies. Adv Funct Mater. (2023) 2303373:1–24. doi: 10.1002/adfm.202303373

[5] Hensel B Pieper L Jung M Schulze M. Influence of age, breed, and season on the quality of boar semen stored at low-temperature. Theriogenology. (2023) 208:102–8. doi: 10.1016/j.theriogenology.2023.06.010, PMID: 37307735

[6] Knecht D Jankowska-Makosa A Duzinski K. The effect of age, interval collection and season on selected semen parameters and prediction of AI boars productivity. Livest Sci. (2017) 201:13–21. doi: 10.1016/j.livsci.2017.04.013

[7] Broekhuijse MLWJ Sostaric E Feitsma H Gadella BM. The value of microscopic semen motility assessment at collection for a commercial artificial insemination center, a retrospective study on factors explaining variation in pig fertility. Theriogenology. (2012) 77:1466–1479.e3. doi: 10.1016/j.theriogenology.2011.11.016, PMID: 22289218

[8] Banaszewska D Kondracki S. An assessment of the breeding maturity of insemination boars based on ejaculate quality changes. Folia Biol. (2012) 60:151–62. doi: 10.3409/fb60_34.151162, PMID: 23342910

[9] Huang YH Lo LL Liu SH Yang TS. Age-related changes in semen quality characteristics and expectations of reproductive longevity in Duroc boars. Anim Sci J. (2010) 81:432–7. doi: 10.1111/j.1740-0929.2010.00753.x, PMID: 20662811

[10] Gunes S Hekim GN Arslan MA Asci R. Effects of aging on the male reproductive system. J Assist Reprod Genet. (2016) 33:441–54. doi: 10.1007/s10815-016-0663-y, PMID: 26867640 PMC4818633

[11] Katz-Jaffe MG Parks J Mccallie B Schoolcraft WB. Aging sperm negatively impacts in vivo and in vitro reproduction: a longitudinal murine study. Fertil Steril. (2013) 100:262–268.e2. doi: 10.1016/j.fertnstert.2013.03.021 23579004

[12] Fraser L Strzezek J Filipowicz K Mogielnicka-Brzozowska M Zasiadczyk L. Age and seasonal-dependent variations in the biochemical composition of boar semen. Theriogenology. (2016) 86:806–16. doi: 10.1016/j.theriogenology.2016.02.035, PMID: 27114169

[13] Czubaszek M Andraszek K Banaszewska D. Influence of the age of the individual on the stability of boar sperm genetic material. Theriogenology. (2020) 147:176–82. doi: 10.1016/j.theriogenology.2019.11.018, PMID: 31767186

[14] Abdelmoughit Badi AB El Khalil K Allai L Essamadi A Nasser B El Amiri B. Does advanced age affect reproductive variables, semen composition, and liquid semen storage during different seasons in Boujaâd rams? Theriogenology. (2018) 197:40–7. doi: 10.1016/j.anireprosci.2018.08.004 30143278

[15] Fuerst-Waltl B Schwarzenbacher H Perner C Solkner J. Effects of age and environmental factors on semen production and semen quality of Austrian Simmental bulls. Anim Reprod Sci. (2006) 95:27–37. doi: 10.1016/j.anireprosci.2005.09.002, PMID: 16207516

[16] Preston BT Saint Jalme M Hingrat Y Lacroix F Sorci G. The sperm of aging male bustards retards their offspring’s development. Nat Commun. (2015) 6:6146. doi: 10.1038/ncomms7146 25647605 PMC4338826

[17] Vuarin P Lesobre L Leveque G Saint Jalme M Lacroix F Hingrat Y. Paternal age negatively affects sperm production of the progeny. Ecol Lett. (2021) 24:719–27. doi: 10.1111/ele.13696, PMID: 33565248

[18] Brito MM Angrimani DSR Lucio CF Vannucchi CI. A case trial study of the effect of ageing on fresh and post-thaw sperm in dogs. Andrologia. (2018) 50:e13123. doi: 10.1111/and.13123, PMID: 30105824

[19] De La Fuente-Lara A Hesser A Christensen B Gonzales K Meyers S. Effects from aging on semen quality of fresh and cryopreserved semen in labrador retrievers. Theriogenology. (2019) 132:164–71. doi: 10.1016/j.theriogenology.2019.04.013, PMID: 31029847

[20] Paoli D Pecora G Pallotti F Faja F Pelloni M Lenzi A. Cytological and molecular aspects of the ageing sperm. Hum Reprod. (2019) 34:218–27. doi: 10.1093/humrep/dey357, PMID: 30551142

[21] Rosiak-Gill A Gill K Jakubik J Fraczek M Patorski L Gaczarzewicz D. Age-related changes in human sperm DNA integrity. Aging. (2019) 11:5399–411. doi: 10.18632/aging.102120, PMID: 31412318 PMC6710060

[22] Tsakmakidis IA Khalifa TA Boscos CM. Age-related changes in quality and fertility of porcine semen. Biol Res. (2012) 45:381–6. doi: 10.4067/S0716-97602012000400009, PMID: 23558995

[23] Everett RW Bean B. Environmental influences on semen output. J Dairy Sci. (1982) 65:1303–10. doi: 10.3168/jds.S0022-0302(82)82344-8 7108021

[24] Snoj T Kobal S Majdic G. Effects of season, age, and breed on semen characteristics in different breeds in a 31-year retrospective study. Theriogenology. (2013) 79:847–52. doi: 10.1016/j.theriogenology.2012.12.014, PMID: 23380262

[25] Taylor JF Bean BH Marshall CE Sullivan JJ. Genetic and environmental components of semen production traits of artificial insemination Holstein bulls. J Dairy Sci. (1985) 68:2703–22. doi: 10.3168/jds.S0022-0302(85)81155-3

[26] Ahmad E Ahmad N Naseer Z Aleem M Khan MS Ashiq M. Relationship of age to body weight, scrotal circumference, testicular ultrasonograms, and semen quality in Sahiwal bulls. Trop Anim Health Prod. (2011) 43:159–64. doi: 10.1007/s11250-010-9668-1, PMID: 20680443

[27] Boujenane I Boussaq K. Environmental effects and repeatability estimates for sperm production and semen quality of Holstein bulls. Arch Anim Breed. (2013) 56:971–9. doi: 10.7482/0003-9438-56-098

[28] Fraser L Strzezek J Kordan W. Post-thaw sperm characteristics following long-term storage of boar semen in liquid nitrogen. Anim Reprod Sci. (2014) 147:119–27. doi: 10.1016/j.anireprosci.2014.04.010, PMID: 24819551

[29] Li JW Parrilla I Ortega MD Martinez EA Rodriguez-Martinez H Roca J. Post-thaw boar sperm motility is affected by prolonged storage of sperm in liquid nitrogen. A retrospective study. Cryobiology. (2018) 80:119–25. doi: 10.1016/j.cryobiol.2017.11.004, PMID: 29146065

[30] Amaral A Lourenco B Marques M Ramalho-Santos J. Mitochondria functionality and sperm quality. Reproduction. (2013) 146:R163–74. doi: 10.1530/REP-13-0178 23901129

[31] Amaral S Amaral A Ramalho-Santos J. Aging and male reproductive function: a mitochondrial perspective. Front Biosci. (2013) 5:181–97. doi: 10.2741/S365, PMID: 23277044

[32] Chianese R Pierantoni R. Mitochondrial reactive oxygen species (ROS) production alters sperm quality. Antioxidants. (2021) 10:92. doi: 10.3390/antiox10010092 33440836 PMC7827812

[33] Moraes CR Meyers S. The sperm mitochondrion: organelle of many functions. Anim Reprod Sci. (2018) 194:71–80. doi: 10.1016/j.anireprosci.2018.03.024 29605167

[34] Guthrie HD Welch GR. Determination of intracellular reactive oxygen species and high mitochondrial membrane potential in Percoll-treated viable boar sperm using fluorescence-activated flow cytometry. J Anim Sci. (2006) 84:2089–100. doi: 10.2527/jas.2005-766, PMID: 16864869

[35] Peña FJ Johannisson A Wallgren M Rodríguez-Martínez H. Assessment of fresh and frozen-thawed boar semen using an Annexin-V assay: a new method of evaluating sperm membrane integrity. Theriogenology. (2003) 60:677–89. doi: 10.1016/S0093-691X(03)00081-5, PMID: 12832017

[36] Castillo J Jodar M Oliva R. The contribution of human sperm proteins to the development and epigenome of the preimplantation embryo. Hum Reprod Update. (2018) 24:535–55. doi: 10.1093/humupd/dmy017, PMID: 29800303

[37] Daigneault BW. Dynamics of paternal contributions to early embryo development in large animals. Biol Reprod. (2021) 104:274–81. doi: 10.1093/biolre/ioaa182, PMID: 32997138

[38] Ashapkin V Suvorov A Pilsner JR Krawetz SA Sergeyev O. Age-associated epigenetic changes in mammalian sperm: implications for offspring health and development. Hum Reprod Update. (2023) 29:24–44. doi: 10.1093/humupd/dmac033, PMID: 36066418 PMC9825272

