# Supplementary figures and images for: Effects of different ages on frozen semen quality and in vitro fertilization efficiency in Wannan black pigs
Source: Front Vet Sci. 2024 May 31;11:1395718. doi: 10.3389/fvets.2024.1395718 (PMC11177872; doi:10.3389/fvets.2024.1395718)

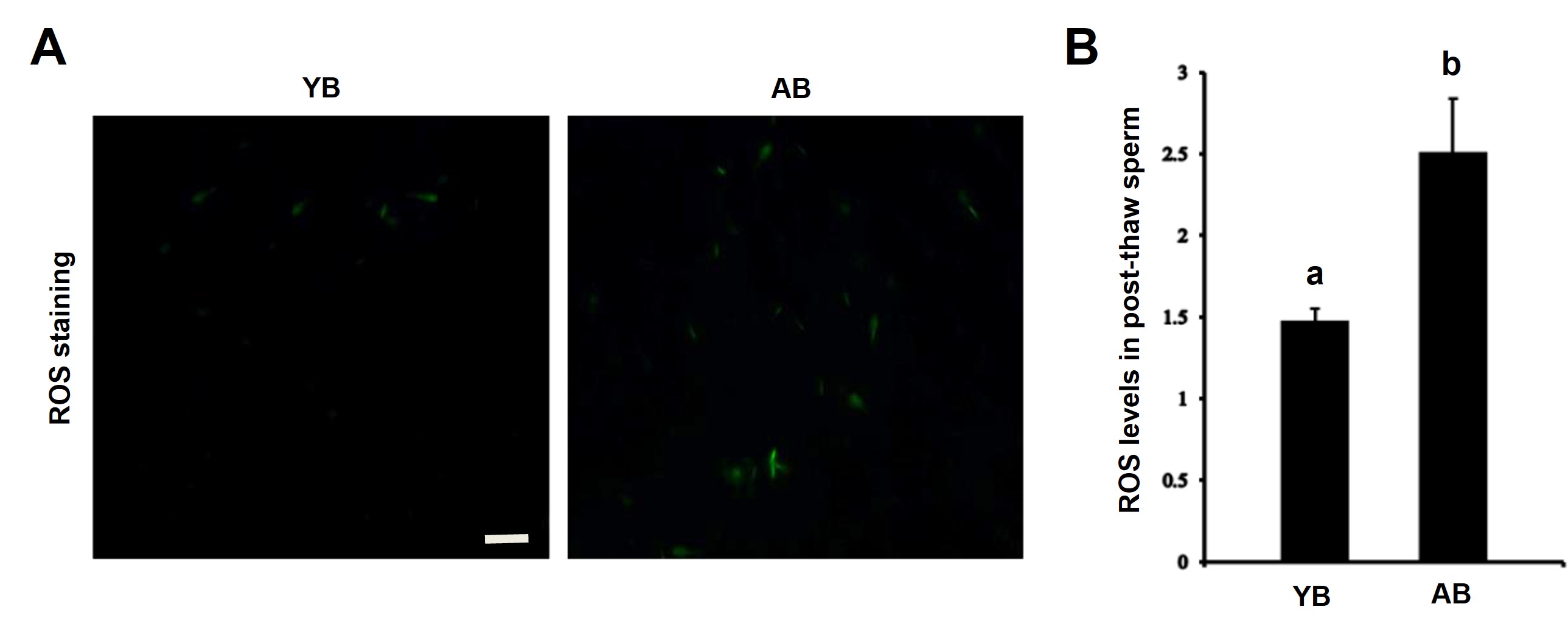

Supplement: FIGURE S1 — Analysis of ROS levels of frozen-thawed sperm between young and aged boars. (A) Representative images of ROS staining in frozen-thawed sperm between YB and AB. (B) ROS levels in frozen-thawed sperm between YB and AB. The fluorescence intensity of ROS was measured The fluorescence intensity of ROS was measured by the confocal microscopy using identical settings and parameters. The data are presented as mean ± S.E.M and different letters on the bars indicate significant differences (p < 0.05). The data are presented as mean ± S.E.M and different letters on the bars indicate significant differences (p < 0.05). [file Image_1.jpeg]
